# Haemolytic uraemic syndrome - a rare case report of bloody diarrhoea in adults

**DOI:** 10.1186/s12876-019-1071-4

**Published:** 2019-08-28

**Authors:** Shiva Thambiah Radhakrishnan, Aruchuna Ruban, Aarthy Kanmany Uthayakumar, Patrizia Cohen, Jeremy Levy, Julian Teare

**Affiliations:** 1 Department of Gastroenterology, Imperial College Healthcare NHS trust, St Marys Hospital, London, W2 1NY England; 2 Department of Surgery and Cancer, Imperial College London, W2 1NY London, England; 3 University College Hospital, 235 Euston Road, Bloomsbury, London, NW1 2BU England; 4 Imperial College Healthcare NHS trust, St Marys Hospital, London, W2 1NY England; 5 Imperial College Healthcare NHS Trust, Hammersmith hospital, London, W12 0HS England

**Keywords:** Bloody diarrhoea, Haemolytic uraemic syndrome, *E*.*coli* 0157, Plasma exchange, Shiga-toxin

## Abstract

**Background:**

Haemolytic uraemic syndrome is a rarely seen in adults often leading to critical illness. This case highlights how difficult it can be to establish a diagnosis and treat when a patient presents with bloody diarrhoea.

**Case presentation:**

A 17-year-old Iraqi man presented to the emergency department with abdominal pain and bloody diarrhoea. He was initially treated as acute appendicitis, undergoing an appendectomy but following a recurrence in his symptoms a colonoscopy was performed. A diagnosis of shiga toxin-producing *Escherichia coli* leading to HUS was suspected following histology obtained at colonoscopy and this was confirmed on antibody testing. Despite intravenous fluids and supportive therapy the patient’s symptoms and condition deteriorated. He developed seizures and acute renal failure requiring intubation and plasma exchange in the intensive care setting. He eventually required treatment with ecluzimab therapy; a monoclonal antibody and subsequently made a full recovery.

**Conclusions:**

Haemolytic uraemic syndrome is a triad of progressive renal failure, thrombocytopenia and haemolytic anaemia which is a condition rarely seen in adults. It is usually associated with an *E. coli* infection and supportive therapy remains the mainstay of treatment.

## Background

Infection with *E.coli 0157* can present with a variety of symptoms including bloody diarrhoea and abdominal cramps. The bacterium is commonly transferred by a feco-oral route and undercooked meat is a known culprit [1]. This infection is linked to Haemolytic uraemic syndrome (HUS) which presents with the triad of progressive renal failure, thrombocytopenia and haemolytic anaemia. HUS can be classified as either typical (diarrhoea associated) or atypical (non-diarrhoea associated such as following a urinary tract infection), [2]. We present a case of a 17-year-old male with bloody diarrhoea who proceeded to be affected by severe HUS including neurological sequalae. Our case highlights the difficulties in establishing a diagnosis and treatment when a patient presents with bloody diarrhoea.

## Case presentation

A 17-year-old Iraqi male presented to the emergency department with a 2 day history of right iliac fossa pain, vomiting and a few episodes of diarrhoea. A clinical diagnosis of appendicitis was made and he was treated with intravenous antibiotics and underwent an appendicectomy, the histology of which was normal. At the time of the operation, the surgeon noted the right colon appeared to be inflamed. The patient had no prior medical history and his family history was nil of note.

A computed tomography (CT) scan was performed postoperatively which showed thickening of the ascending colon with some submucosal oedema in the caecum with associated local regional lymph nodes. However the patient’s condition improved enough to be sent home the day after his operation.

Two days later he returned to hospital with bloody diarrhoea up to ten times a day, with associated fever and a tender abdomen. Baseline admission investigations are shown in Table 1. Initial stool cultures were negative including for *Escherichia coli* 0157 (*E. coli*), and the patient was started on intravenous cefuroxime and metronidazole. An abdominal radiograph did not reveal colonic dilatation. A colonoscopy was performed which showed patchy pan-colitis, maximal in the ascending and descending colon.

**Table 1:** Baseline admission investigations

	Measurement	Normal Range
Haemoglobin	182 g/L	130–168 g/L
Mean cell volume	88.2 fL	83.5–99.5 fL
Platelets	332 × 10^9^/L	130–370 × 10^9^/L
White cell count	12.4 × 10^9^/L	4.2–10.6 × 10^9^/L
Neutrophils	7.7 × 10^9^/L	2.0–7.1 × 10^9^/L
Lymphocytes	3.4 × 10^9^/L	1.1–3.6 × 10^9^/L
Sodium	141 mmol/L	133–146 mmol/L
Potassium	4.1 mmol/L	3.5–5.3 mmol/L
Urea	3.6 mmol/L	2.5–7.8 mmol/L
Creatinine	68 μmol/L	60–125 μmol/L
Bilirubin	9 μmol/L	0–21 μmol/L
Alkaline phosphatase	106 IU/L	60–370 IU/L
Alanine aminotransferase	15 IU/L	0–40 IU/L
Abdominal radiograph	No toxic megacolon	

**Fig. 1:**
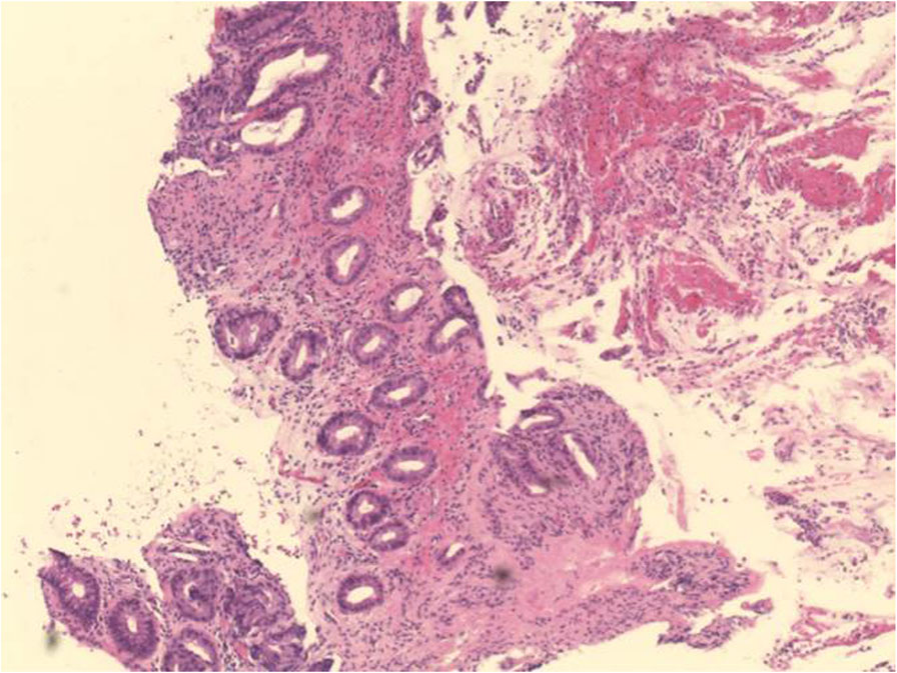
“Large bowel mucosa showing withered atrophic crypts near the surface and lamina propria hyalinisation along with haemorrhage. Free lying pseudomembranous exudate composed of mucus, inflammatory exudate and blood is noted near the luminal surface. These findings are highly suggestive of enterohaemorrhagic *E.coli”*

Given the age of the patient and location of his symptoms, plus the initial findings on the CT scan, a diagnosis of inflammatory bowel disease was considered. The other main differential was an infective colitis caused by Campylobacter, Shigella, *E. coli* or *Clostridium difficile*. Tuberculosis (TB) was another possibility and although the patient was originally from Iraq there was no recent history of foreign travel or relevant contact history of TB exposure. The appearances at colonoscopy were most suggestive of Crohn’s disease so the patient was commenced on intravenous hydrocortisone. Colonic biopsies were taken with the histology results shown in Fig. 1.

Despite 24 h of steroid therapy, the patient still had on-going bloody diarrhoea although his pain had now settled. His renal function had declined from a normal baseline to a serum urea of 11.3 mmol/L and a serum creatinine of 170 μmol/L, and despite being adequately hydrated with intravenous fluids he became anuric. He was also noted to be thrombocytopenic with a platelet count of 81. Additionally, the patient became anaemic and his blood film showed Red cell fragments: 10 fragments per × 40 objective field, indicating haemolysis. Clinically the patient became disorientated and agitated and he rapidly deteriorated developing partial-complex seizures. This rapid deterioration required intubation and he was promptly admitted to the intensive care unit (ITU). A diagnosis of typical Haemolytic Uraemic Syndrome (HUS) was made following the history of gastrointestinal illness coupled with an acute kidney injury and thrombocytopenia. His levels of ADAMTS13 were normal, making a diagnosis of Thrombotic Thrombocytopenic Purpura (TTP) less likely. The patient received haemofiltration and plasma exchange with fresh frozen plasma but showed little signs of improvement so after 10 days he was commenced on Eculizumab a monoclonal antibody. He remained on the ITU for a further 35 days and spent a further 28 days on renal high-dependency unit during which time he gradually improved. He remained seizure free with normalisation of his platelets and biochemical profile and was discharged well from hospital.

## Discussion and conclusions

HUS is a rare condition characterized by progressive renal failure, thrombocytopenia and haemolytic anaemia. It can be classified as either typical (diarrhoea associated) or atypical (non-diarrhoea associated such as following a urinary tract infection), [2]. A well-recognised cause of HUS is due to infection with shiga toxin-producing bacteria, the most common of which is *E.coli* O157:H7, [3]. This mainly occurs in childhood, and is characterised by a prodromal acute gastroenteritis followed by haemolytic anaemia, thrombocytopenia and acute kidney injury.

Shiga toxin releasing *E.coli* (STEC) gastroenteritis can be difficult to diagnose as patients may not present with all the hallmark features. Bloody diarrhoea can occur a median of 3 days after ingestion of contaminated food and some patients may also report to suffer from severe abdominal pain and painful defecation, [4]. The latter may help to distinguish STEC from other causes of bacterial gastroenteritis where these symptoms would be unusual, [5].

The primary pathophysiological mechanism in typical HUS is vascular endothelial cell injury by both inflammatory and non-inflammatory mechanisms, such as cytokine release, [2]. Renal dysfunction in HUS is thought to be caused by microthrombi of platelets and fibrin in arterioles and capillaries, [6]. Approximately 40% of patients with STEC require renal replacement therapy, and of these, 20% will have permanent renal dysfunction, [7]. Coagulopathy and thrombocytopenia occur due to increased platelet consumption. Fragmented red cells, a key feature of HUS, is due to mechanical damage from shear stress in fibrin lined vessels as well as peroxidative damage.

Our patient did have microangiopathic haemolysis, acute renal failure, thrombocytopenia and was eventually found to be STEC antibody positive, which confirmed a diagnosis of typical HUS, [8]. The cause of his seizures was unclear but neurological involvement in HUS is a recognized complication and is the most frequent cause for fatalities, [7]. The pathophysiology is thought to be multifactorial and still poorly understood but may be due to a combination of microinfarctions in key anatomical regions such as the brainstem or the effect of increased inflammatory cytokines seen to be present in higher concentrations in patients with neurological complications such as seizures and encephalopathy, [9].

Neurological complications are also a clinical feature of thrombotic TTP and both TTP and HUS are considered to be on a spectrum of thrombotic microangiopathies, [10]. Though TTP shares a similar clinical picture, in this syndrome there is a deficiency or formation of antibodies to ADAMTS13, a von Willebrand cleaving protease which helps to differentiate the two conditions, [11].

Supportive therapy remains the mainstay of treatment in HUS with adequate fluid rehydration where required, [12]. The role of antibiotics is controversial in STEC gastroenteritis with some evidence that antibiotic therapy may be detrimental triggering further release of shiga toxin by bacterial lysis [13–15], which may explain the delayed deterioration in our patient.

A likely contribution to the pathogenesis of STEC-HUS is activation of the alternative complement pathway and so patients with life threatening complications may receive benefit with short term therapy with ecluzimab, a monoclonal antibody against C5, which inhibits terminal complement complex formation. This has already been reported as an effective treatment in atypical HUS where the role of the complement pathway is well established, [5, 15, 16]. In a case series following an outbreak of STEC (*E.coli* O104:H4) in Germany, rapid clinical improvement following eculizumab was reported, [17, 18]. All patients had renal failure and neurological involvement which had not responded to plasma exchange. Due to the cost of the drug, its use is limited to only the most severe cases and therefore treatment is often delayed, [19]. Within a paediatric population, early treatment of HUS with eculizimab in patients with neurological complications led to better outcomes, compared to delayed therapy initiation in patients with rapidly progressive HUS, [20].

Plasma exchange therapy in typical HUS is controversial in adults as there is a lack of evidence for its efficacy but it is used in some cases where severe neurological abnormalities exist, [21, 22]. In addition, immunoadsorption with intravenous immunoglobulins (IVIG) can also be used in this setting and has been shown to lead to improvement [23]. The rationale is based on a suspected immune-mediated mechanism to neurological sequelae, which explains the delayed onset of these symptoms in disease course.

## Learning points

- HUS secondary to STEC is well described condition in the paediatric population but its incidence and epidemiology in adults is relatively unknown.
- The classic triad in HUS is haemolytic anaemia, acute renal failure and thrombocytopenia. Early checking of ADAMTS13 and for STEC is advised if indicated.
- Antibiotics are relatively contraindicated in *E.coli* associated HUS.
- The role of the histopathologist is crucial in the recognition of E.Coli as this can speed up diagnosis and onward management decisions.
- Treatment is largely supportive with fluid resuscitation and dialysis if required, although more recently ecluzimab and immunoadsorption provide additional treatment options for patients who are not clinically improving.

## Data Availability

Not applicable. Reference list attached above.

## Abbreviations

CT: Computed tomography
*E.coli*: *Escherichia coli*
HUS: Haemolytic uraemic syndrome
ITU: intensive care unit
IVIG: intravenous immunoglobulins
STEC: Shiga toxin releasing *E.coli*
TB: Tuberculosis
TTP: Thrombotic Thrombocytopenic Purpura

## References

[1] Su C, Brandt LJ (1995). *Escherichia coli* O157: H7 infection in humans. Ann Intern Med.

[2] Kaplan BS, Meyers KE, Schulman SL (1998). The pathogenesis and treatment of hemolytic uremic syndrome. J Am Soc Nephrol.

[3] Tarr PI, Gordon CA, Chandler WL. Shiga-toxin-producing *Escherichia coli* and haemolytic uraemic syndrome*. Lancet 2*005;25;365(9464):1073–86. 10.1016/S0140-6736(05)71144-2 15781103

[4] Bell Beth P. (1994). A Multistate Outbreak of Escherichia coli O157:H7—Associated Bloody Diarrhea and Hemolytic Uremic Syndrome From Hamburgers. JAMA.

[5] Slutsker Laurence (1997). Escherichia coli O157: H7 Diarrhea in the United States: Clinical and Epidemiologic Features. Annals of Internal Medicine.

[6] Fakhouri Fadi, Zuber Julien, Frémeaux-Bacchi Véronique, Loirat Chantal (2017). Haemolytic uraemic syndrome. The Lancet.

[7] Trachtman Howard, Austin Catherine, Lewinski Maria, Stahl Rolf A. K. (2012). Renal and neurological involvement in typical Shiga toxin-associated HUS. Nature Reviews Nephrology.

[8] Kavanagh D, Goodship TH, Richards A (2013). Atypical hemolytic uremic syndrome. Sem Nephrol.

[9] SHIRAISHI M, ICHIYAMA T, MATSUSHIGE T, IWAKI T, IYODA K, FUKUDA K, MAKATA H, MATSUBARA T, FURUKAWA S (2008). Soluble tumor necrosis factor receptor 1 and tissue inhibitor of metalloproteinase-1 in hemolytic uremic syndrome with encephalopathy. Journal of Neuroimmunology.

[10] Kappler Shane, Ronan-Bentle Sarah, Graham Autumn (2014). Thrombotic Microangiopathies (TTP, HUS, HELLP). Emergency Medicine Clinics of North America.

[11] Haspel RL, Jarolim P (2005). The “cutting” edge: von Willebrand factor-cleaving protease activity in thrombotic microangiopathies. Transfus Apheresis Sci.

[12] Loirat C, Saland J, Bitzan M (2012). Management of hemolytic uremic syndrome. Presse Med.

[13] Thomas DE, Elliot EJ (2013). Interventions for preventing diarrhea-associated hemolytic uremic syndrome: systematic review. BMC Public Health.

[14] Wong Craig S., Jelacic Srdjan, Habeeb Rebecca L., Watkins Sandra L., Tarr Phillip I. (2000). The Risk of the Hemolytic–Uremic Syndrome after Antibiotic Treatment ofEscherichia coliO157:H7 Infections. New England Journal of Medicine.

[15] Dundas S, Todd WA, Stewart AI, Murdoch PS, Chaudhuri AK, Hutchinson SJ (2001). The Central Scotland Escherichia coli O157: H7 outbreak: risk factors for the hemolytic uremic syndrome and death among hospitalized patients. Clin Infect Dis.

[16] Orth D, Würzner R (2010). Complement in typical hemolytic uremic syndrome. Sem Thromb Hemost.

[17] Lapeyraque Anne-Laure, Malina Michal, Fremeaux-Bacchi Véronique, Boppel Tobias, Kirschfink Michael, Oualha Mehdi, Proulx François, Clermont Marie-José, Le Deist Françoise, Niaudet Patrick, Schaefer Franz (2011). Eculizumab in Severe Shiga-Toxin–Associated HUS. New England Journal of Medicine.

[18] JT Kielstein G, Beutel SF (2012). Best supportive care and therapeutic plasma exchange with or without eculizumab in Shiga-toxin-producing *E. coli* O104:H4 induced haemolytic–uraemic syndrome: an analysis of the German STEC-HUS registry. Nephrol Dial Transplant.

[19] Ekinci Zelal, Bek Kenan, Aytac Mehmet Baha, Karadenizli Aynur, Hancer Veysel Sabri (2014). Renal outcome with eculizumab in two diarrhea-associated hemolytic–uremic syndrome cases with severe neurologic involvement. Hong Kong Journal of Nephrology.

[20] Pape Lars, Hartmann Hans, Bange Franz Christoph, Suerbaum Sebastian, Bueltmann Eva, Ahlenstiel-Grunow Thurid (2015). Eculizumab in Typical Hemolytic Uremic Syndrome (HUS) With Neurological Involvement. Medicine.

[21] Tarr PI, Karpman D (2012). Editorial commentary: Escherichia coli O104: H4 and hemolytic uremic syndrome: the analysis begins. Clin Infect Dis.

[22] Dundas S, Murphy J, Soutar RL, Jones GA, Hutchinson SJ, Todd WTA (1999). Effectiveness of therapeutic plasma exchange in the 1996 Lanarkshire Escherichia coli O157:H7 outbreak. The Lancet.

[23] Greinacher A, Friesecke S, Abel P (2011). Treatment of severe neurological deficits with IgG depletion through immunoadsorption in patients with *Escherichia coli* O104:H4-associated haemolytic uraemic syndrome: a prospective trial. Lancet.

